# The Role of Acupuncture in Relieving Post-Hemorrhoidectomy Pain: A Systematic Review of Randomized Controlled Trials

**DOI:** 10.3389/fsurg.2022.815618

**Published:** 2022-03-28

**Authors:** Huan Chen, Weina Zhang, Yuanjie Sun, Ruimin Jiao, Zhishun Liu

**Affiliations:** ^1^Department of Acupuncture, Guang'anmen Hospital, China Academy of Chinese Medical Sciences, Beijing, China; ^2^Tianjin University of Traditional Chinese Medicine, Tianjin, China; ^3^Beijing University of Chinese Medicine, Beijing, China; ^4^Institute of Acupuncture and Moxibustion, China Academy of Chinese Medical Science, Beijing, China

**Keywords:** acupuncture, electro-acupuncture (EA), manual acupuncture, post-hemorrhoidectomy pain, systematic review

## Abstract

**Background:**

Post-hemorrhoidectomy pain (PHP) remains one of the complications of hemorrhoidectomy and can delay patient's recovery. Current clinical guideline on PHP remains skeptical on the effectiveness of acupuncture, which has been applied for PHP in practice with inconsistent evidence.

**Objectives:**

This systematic review aimed to evaluate the effectiveness of acupuncture on PHP by reviewing existing evidence.

**Methods:**

Nine databases such as PubMed and Embase were searched for randomized controlled trials (RCTs) from inception to 30th September 2021. The outcome measures on pain level after hemorrhoidectomy, dose of rescue analgesic drug used, quality of life, adverse events, etc., were extracted and analyzed in a narrative approach.

**Results:**

Four RCTs involving 275 patients were included in the analysis. One study showed that the visual analog scale (VAS) score was significantly lower in the electro-acupuncture (EA) group compared to that in the sham acupuncture (SA) group at 6, 24 h after surgery and during the first defecation (*p* < 0.05). Similar trends were found in the verbal rating scale (VRS) and Wong-Baker Faces scale (WBS) score but at different time points. Another study also found EA was effective on relieving pain during defecation up to 7 days after surgery when compared with local anesthetics (*p* < 0.05). However, two studies evaluating manual acupuncture (MA) compared with active medications for PHP showed inconsistent results on effectiveness. Variability was found in the quality of included studies.

**Conclusions:**

Although benefit of acupuncture on PHP, especially EA on defecation after surgery, was observed at some time points, evidence on effectiveness of acupuncture on PHP was not conclusive.

**Systematic Review Registration:**

https://www.crd.york.ac.uk/PROSPERO/, PROSPERO, identifier: CRD42018099961.

## Introduction

Hemorrhoids are the most common proctological diseases with varied prevalence globally (1–4). It is estimated that more than 50% of the US population over 50 years old experienced hemorrhoid problems (5). While in China the estimated prevalence is 49.14% (6). Hemorrhoidectomy is an effective treatment, especially for hemorrhoids at grade III or IV (3). However, post-hemorrhoidectomy pain (PHP), especially pain during defecation, remains as one of the most important patient complaints after surgery (7) and appears to be multifactorial such as spasm within the internal sphincter, insertion of anal pack, damage of nerve endings, and mucosal injury (8, 9), and it also depends on the types of surgical procedures, individual tolerance, and type and regimen of postoperative analgesia (10).

It is recommended that multiple analgesic measures, such as medication, physical, and cognitive-behavioral therapies, should be taken for PHP to reduce the amounts of narcotics used and to speed up the recovering process (11). However, conventional analgesic methods, such as nonsteroidal anti-inflammatory drugs (NSAIDs), paracetamol, and opioids, are effective in addressing pain, but often have side effects, such as dizziness, nausea, vomiting, constipation, and even intolerance, which might disrupt proper recovery and lead to a poor prognosis (12–14). In addition, the levels of evidences for physical and cognitive-behavioral therapies are weak.

Previous evidence (15, 16) and meta-analysis (17) revealed that acupuncture can be applied as a single anesthetic technique in operation, such as craniotomy, while recent studies added that acupuncture could be beneficial as a supplement to relieve post-operative pain, including PHP (18–21). However, evidences on effect size and timing and regimen of acupuncture on PHP remain scarce, and no systematic review has been found on this topic so far. The aim of this study is to address the knowledge gap by reviewing existing evidences and evaluating the effectiveness of acupuncture on PHP.

## Methods

The protocol of this systematic review was registered at PROSPERO (https://www.crd.york.ac.uk/PROSPERO/) (registration number—CRD42018099961). This systematic review was performed according to the Preferred Reporting Items for Systematic Review and Meta-Analysis (PRISMA) statement (22).

### Search Strategies and Study Selection

We searched the following nine databases from inception to 30th September 2021: PubMed, Embase, the Cochrane Library, Medical Online, Korea Science, China National Knowledge Infrastructure (CNKI), Chinese Biomedical database (CBM), Chinese Scientific Journal database (VIP), and the Wan Fang database. The keywords for search strategy included acupuncture, postoperative pain, hemorrhoid, and RCT, and tailored search strategy for each database is shown in Appendix 1.

Studies were included if they: (1) were randomized controlled trials (RCTs) evaluating the effectiveness of acupuncture for postoperative pain of hemorrhoidectomy; (2) were studies looking at patients with PHP (3, 23) regardless of the type of hemorrhoid surgery, age, sex, and race; (3) defined acupuncture in at least one treatment group as penetration of the skin with needles, either combined with other active medication for PHP or not; (4) included no treatment, sham/placebo acupuncture, or active medication, etc., as comparison; (5) reported outcomes including but not limited to pain intensity score measured by the visual analog scale (VAS) (24), the verbal rating scale (VRS) (25), and/or the Wong-Baker Faces scale (WBS) (26) at various time points, dose of analgesic drug used, quality of life, and adverse events related to acupuncture.

Studies were excluded if they: (1) were retrospective studies, case reports, reviews, and conference proceedings; (2) were focusing on acupressure or auricular-plaster (defined as kind of maneuver using cowherb seed to press ear acupoints), or comparing acupuncture with herbal medicine or moxibustion; and (3) recruited patients with other perianal diseases, such as anal fissure.

### Data Collection and Analysis

#### Data Extraction and Risk of Bias Assessment

The following information was extracted from papers: authors, year, number of study centers, sample size, patients' sex and age, type of hemorrhoids, type of surgical procedure, interventions, duration of treatment, acupoints, pain scores and other outcome measures, use of rescue analgesic drugs, and adverse events, etc. The study investigators were contacted *via* email to obtain incomplete data if necessary. It was assumed that the score of postoperative pain was assessed at rest, unless otherwise specified by the study.

Data extraction was completed by two independent researchers (WZ and RJ). Any disagreements were solved by discussion or consulting the supervisor's opinion (ZL). According to the Cochrane risk of bias assessment tool (27), the included studies were assessed for the following categories of risk of bias: random sequence generation, allocation concealment, blinding of participants and personnel, blinding of outcome assessment, incomplete outcome data, selective reporting, and other bias. The risk of bias in each category was divided into three levels: low, unclear, and high.

#### Data Analysis

Quantitative analysis was not performed due to limited number of included studies with substantial heterogeneity on design and outcome measures. Instead, the effectiveness of acupuncture was evaluated qualitatively by presenting and articulating characteristics and outcomes of each study, and a *p*-value of < 0.05 was adopted as threshold of statistical significance. Data of pain level measurement and quality of life, such as scores of the VAS, VRS, and WBS, are usually continuous variables, thus mean difference (MD) with 95% *CI* was used to present the relevant result.

## Results

**Figure 1:**
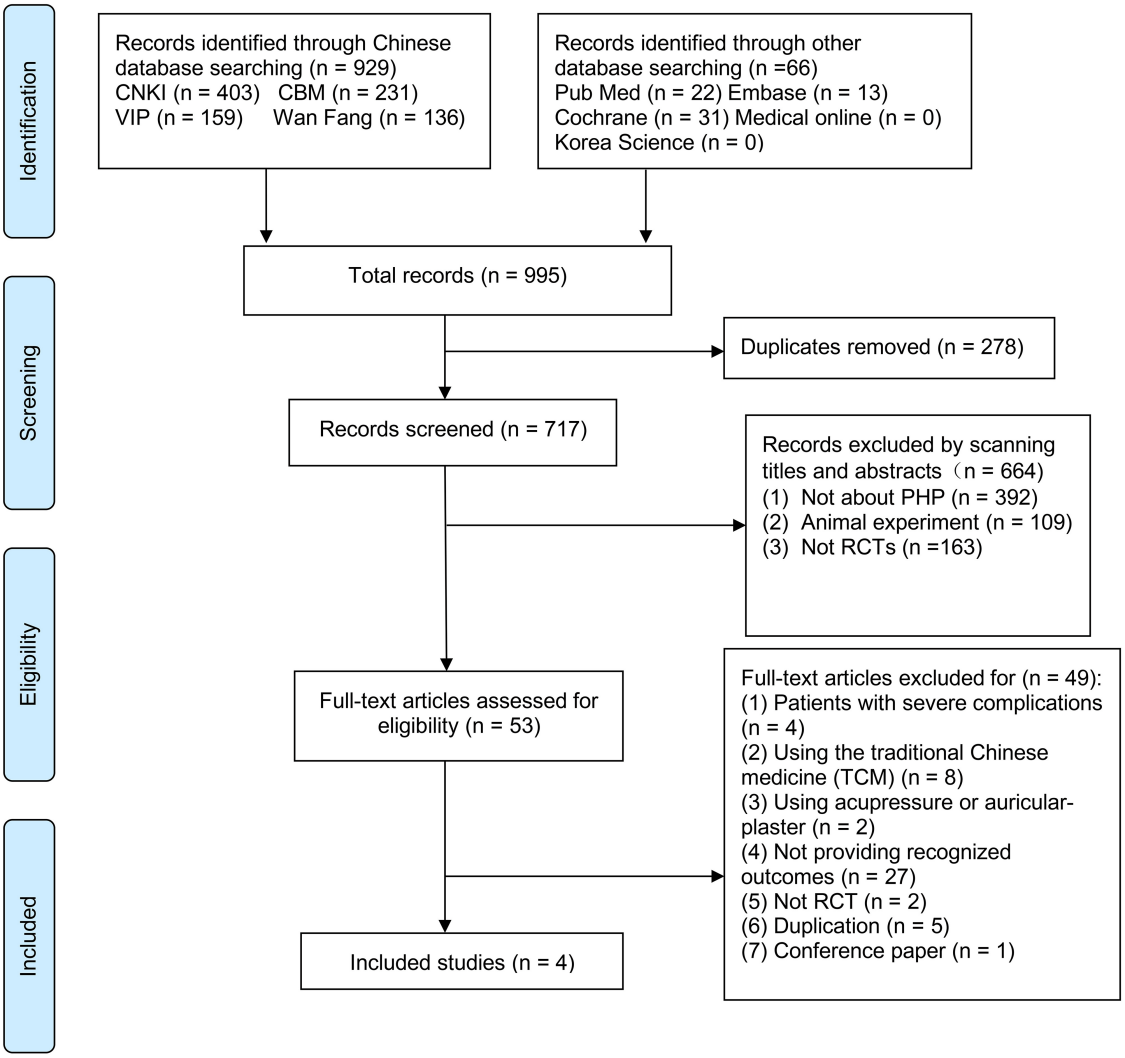
PRISMA flow diagram of Selection of studies process. PRISMA, Preferred reporting items for systematic reviews and meta-analyses; RCT, randomized controlled trials.

A total of 995 studies of acupuncture on PHP were identified in total, of which 278 duplications were removed, and 664 were excluded for not meeting the inclusion criteria after the title and abstract screening, and 49 studies were excluded for meeting the exclusion criteria during the full-text review. The most common reasons for excluding studies were not using recognized outcome measurements for pain (27 studies) and the use of the traditional Chinese medicine in any arm (8 studies). Four studies (20, 28–30) were included eventually. The flow chart of the selection process is presented in Figure 1.

### Characteristics of Included Studies

The four included studies were all RCTs conducted in China, with one study published in English (30) and three (20, 28, 29) in Chinese. The characteristics of included studies are shown in Table 1.

**Table 1:** Characteristics of included randomized controlled trials (RCTs).

**References**	**Sample size (Tx/ Ctrl)**	**Sex (M/F)**	**Age range (Years) (Tx/Ctrl)**	**Type of hemorrhoids**	**Grades**	**Type of surgery**	**Experiment group**				**Control group**		**Outcome measure**	**AE (Tx/Ctrl)**
							**Treatment**	**Depth of needle insertion**	**Frequency/duration**	**Acupoints**	**Treatment**	**Frequency/duration**		
Zeng (28)	65 (35/30)	27/38	22–63/22–63	Mixed	III– IV	Milligan–Morgan	EA (50 Hz)	NR	30mins, once daily 7 days	BL31, BL32, BL33, BL34, BL57, LI4	1% methylene blue injection 2 ml + 0.75% bupivacaine hydrochloride 5 ml; 10 ml	once after surgery	VAS score	Yes/Yes
Xiao et al. (20)	80 (40/40)	46/34	18–75/20–78	Mixed	NR	Milligan–Morgan	MA	NR	30mins, once daily 7 days	EX-UE2, BL30, BL57, GV1, GV2, SP5	Indometacin Suppositories; 0.1 g per time	Once daily 7 days	VAS score	No/Yes
Lin (29)	58 (29/29)	32/26	20–64/19–65	Mixed	NR	Milligan–Morgan	MA	0.5–2cun^a^	30mins, twice daily 3 days	EX-UE2, BL57, GV1, SP6, LR3, LI4	Tramadol hydrochloride tablets; 50 mg per time	Once daily 3 days	VAS score; Dose of analgesic drug	Yes/Yes
Wu et al. (30)	72 (36/36)	33/39	18–50/18–50	Mixed	I–III	Milligan–Morgan	EA (50 Hz)	10–25 mm	30mins, once after surgery	Points around the lesion	Sham acupuncture	30 mins, once after surgery	VAS score; VRS score; WBS score; Quality of life	No/No

*S, single-center; M, multi-center; NR: not reported; Tx: treatment group; Ctrl: control group; M: Male; F: Female; EA: electro-acupuncture; MA: manual acupuncture; AE: adverse event; VAS: visual analog scale; VRS: verbal rating scale; WBS: Wong-Baker Faces scale. ^a^1 cun = 3.33 cm*.

There were 275 patients with PHP enrolled in the four studies with 138 men and 137 women, aged from 18 to 78 years old. One study compared electroacupuncture (EA) with sham acupuncture (SA) (30), and three studies compared electro or manual acupuncture (MA) with active medication (20, 28, 29). Two studies used EA (28, 30) and two used MA (20, 29). EA was described as disposable needles empowered with electric current through electroacupuncture apparatuses to enhance the stimulation to the acupoint in the included studies, while MA as hand maneuver of needles penetrating patient's skin. All of the studies started acupuncture treatment short after hemorrhoidectomy, and the duration and frequency of a single acupuncture session was 30 min once or twice a day, with total durations varied from 1 to 7 days across studies. However, information such as anesthesia technique, duration and extent of surgery, surgeon skill, preexisting pain levels, and perioperative opioid levels, which may affect the estimation of the effect size of acupuncture on PHP, was not provided by included studies except type of surgery technique used (Milligan–Morgan, open hemorrhoidectomy) in all the four studies and patient anxiety level (measured by Symptom Checklist-90 Scale) in one study (30).

Among the four studies, the most frequently used acupoints were Chengshan (BL57, 3 out of 4 studies), Erbai (EX-UE2, 2 studies), Changqiang (GV1, 2 studies), and Hegu (LI4, 2 studies) (Figure 4). All of the four studies reported VAS pain score to measure pain level (20, 28–30), and one study (30) also reported VRS and WBS pain scores and quality of life assessment. One study (29) reported a dose of rescue analgesics used. Adverse events were reported in three studies (20, 28, 29).

### Risk of Bias

**Figure 2:**
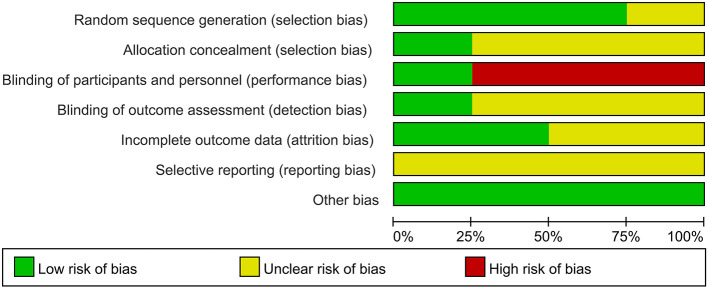
Risk of bias graph.

**Figure 3:**
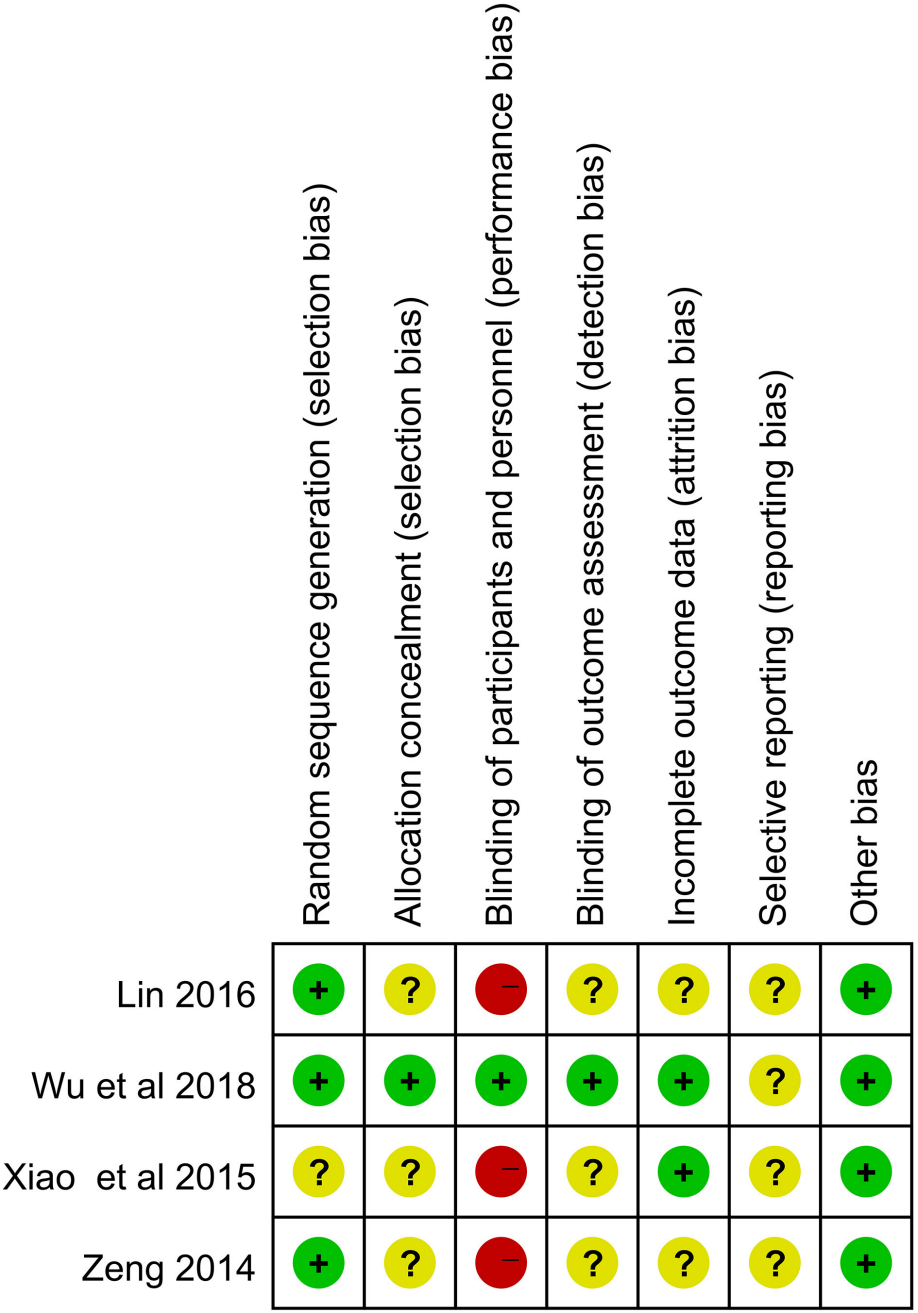
Risk of bias summary.

Three studies (28–30) reported the use of random number table method, but two (28, 29) of them did not report allocation concealment. One study (20) did not report any information on random sequence generation and allocation concealment. One study (30) reported blinding of patient and assessor, and three studies (20, 28, 29) comparing acupuncture with active medicines could not blind patients. Two studies (28, 29) did not report the approach used to deal with missing data. The risks of selective reporting of all included studies were unclear as their protocols were not available to identify any unreported outcomes. No other bias was identified in the review (Figures 2, 3, produced by RevMan 5.2).

**Figure 4:**
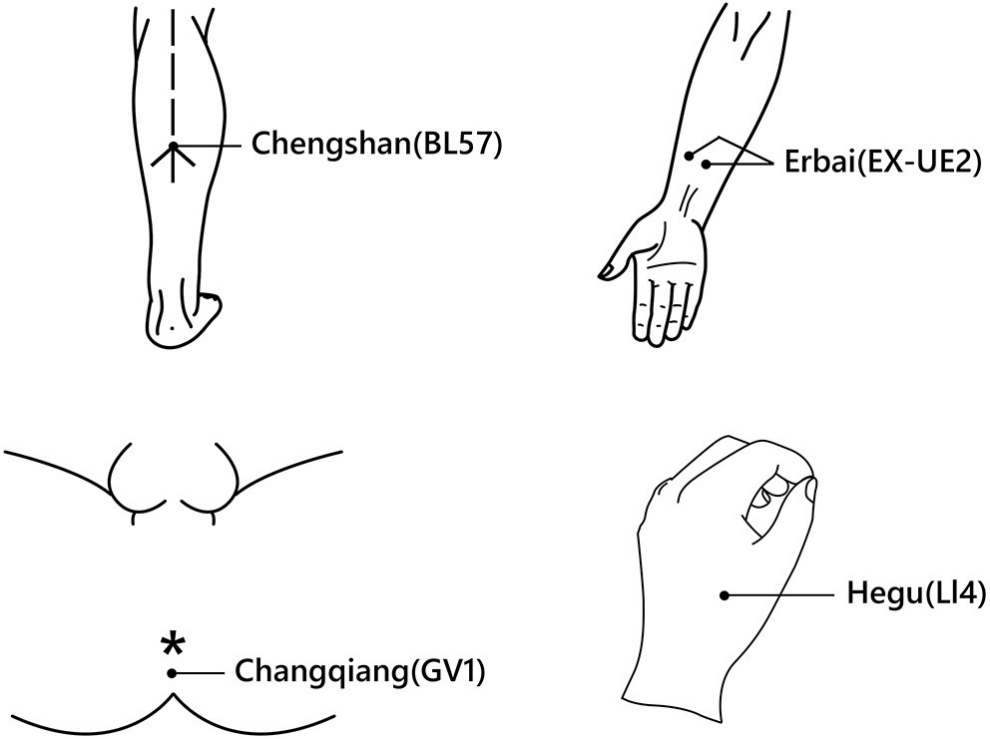
The four most frequently used acupoints. Chengshan (BL57): A point on the Bladder Meridian of Foot-Taiyang, below the juncture of the belly on both sides of the gastrocnemius muscle, on the back of the shank and in the depression formed when the ankle joint stretches out. Erbai (EX-UE2): One point of the Extra Points, on the palmar aspect of the forearm, 4 cun proximal to the transverse crease of the wrist, on each side of the radial flexor muscle tendon of the wrist. Changqiang (GV1): A point on the Governor Vessel, below the tip of the coccyx, at the midway between the tip of the coccyx and the anus. Hegu (LI4): A point on the Large Intestine Meridian of Hand-Taiying, on the dorsum of the hand, between the first and second metacarpal bones, at the middle of the radial side of the second metacarpal bone (31). The picture was drawn and authorized by Biqing Zou. The *demonstrates anus in the figure.

### Effects of Acupuncture on PHP

The detailed results of outcome measures are showed in Table 2.

**Table 2:** Outcomes of included RCTs.

**Studies**	**Outcome measurement**		**Time**	**Experiment group**	**Control group**	***P*-value**
Zeng (28)	VAS score [Mean ± SD]		Baseline	2.67 ± 2.29	2.48 ± 1.90	*p =* 0.719
			at the defecation on 1st day after surgery	5.33 ± 1.43	7.03 ± 1.66	*p* < 0.001
			at the defecation on 2nd day after surgery	4.67 ± 1.79	7.72 ± 0.88	*p* < 0.001
			at the defecation on 3rd day after surgery	3.61 ± 1.78	6.14 ± 1.41	*p* < 0.001
			at the defecation on 5th day after surgery	2.44 ± 1.86	4.69 ± 1.63	*p* < 0.001
			at the defecation on 7th day after surgery	1.61 ± 1.50	3.17 ± 1.73	*p =* 0.003
Xiao et al. (20)	VAS score [Mean ± SD]		Baseline	6.77 ± 1.32	6.69 ± 1.41	*p =* 0.3970
			on the 1st day after surgery	5.75 ± 1.04	6.18 ± 1.12	*p =* 0.0395
			on the 3rd day after surgery	3.87 ± 1.09	5.46 ± 1.14	*p* < 0.001
			on the 5th day after surgery	3.05 ± 1.13	4.63 ± 1.07	*p* < 0.001
			on the 7th day after surgery	2.01 ± 0.96	4.08 ± 0.79	*p* < 0.001
Lin (29)	VAS score [Mean ± SD]		Baseline ^a^	-	-	-
			at 2h after surgery	1.59 ± 0.78	2.07 ± 0.96	*p =* 0.375
			at 4h after surgery	3.55 ± 1.02	3.69 ± 0.97	*p =* 0.917
			at 10h after surgery	2.17 ± 0.84	2.24 ± 0.87	*p =* 0.644
			at 24h after surgery	2.45 ± 0.78	3.07 ± 1.25	*p =* 0.102
			at 48h after surgery	2.10 ± 0.90	2.41 ± 1.12	*p =* 0.046
			at 72h after surgery	2.13 ± 0.79	2.23 ± 0.67	*p =* 0.612
	Dose of rescue analgesic drug used		throughout the study period	50.00 ± 0.00	167.24 ± 30.69	*p =* 0.003
Wu et al. (30)	VAS score [Mean ± SD]		Baseline ^a^	-	-	-
			at 1h after surgery	2.60 ± 1.25	2.87 ± 1.41	*p =* 0.385
			at 2h after surgery	2.44 ± 1.03	2.87 ± 1.28	*p =* 0.121
			at 3h after surgery	2.22 ± 0.98	2.63 ± 0.97	*p =* 0.080
			at 4h after surgery	2.24 ± 0.91	2.59 ± 0.67	*p =* 0.072
			at 5h after surgery	2.17 ± 0.89	2.46 ± 0.63	*p =* 0.115
			at 6h after surgery	2.11 ± 0.85	2.49 ± 0.58	*p =* 0.033
			at 7h after surgery	2.23 ± 0.82	2.62 ± 0.93	*p =* 0.059
			at 8h after surgery	2.35 ± 0.93	2.81 ± 1.06	*p =* 0.053
			at 24h after surgery	1.08 ± 0.66	1.51 ± 0.66	*p =* 0.006
			at 48h after surgery	0.79 ± 0.53	0.86 ± 0.48	*p =* 0.563
			at the first defecation after surgery	1.22 ± 0.64	1.89 ± 0.88	*p* < 0.001
	VRS score [χ^2^]		Baseline^a^^,^^b^	-	-	
			at 1h after surgery	0.53		*p =* 0.468
			at 2h after surgery	0.99		*p =* 0.320
			at 3h after surgery	3.55		*p =* 0.060
			at 4h after surgery	4.47		*p =* 0.035
			at 5h after surgery	1		*p =* 0.317
			at 6h after surgery	0		*p =* 1
			at 7h after surgery	1.27		*p =* 0.260
			at 8h after surgery	1.78		*p =* 0.182
			at 24h after surgery	2.11		*p =* 0.147
			at 48h after surgery	0.24		*p =* 0.626
			at the first defecation after surgery	6.00		*p =* 0.014
	WBS score [χ^2^]		Baseline^a^^,^^b^	-		-
			at 1h after surgery	0.97		*p =* 0.326
			at 2h after surgery	1.01		*p =* 0.316
			at 3h after surgery	2.26		*p =* 0.133
			at 4h after surgery	3.79		*p =* 0.051
			at 5h after surgery	5.69		*p =* 0.017
			at 6h after surgery	3.27		*p =* 0.070
			at 7h after surgery	6.44		*p =* 0.011
			at 8h after surgery	6.04		*p =* 0.014
			at 24h after surgery	2.54		*p =* 0.111
			at 48h after surgery	0.81		*p =* 0.806
			at the first defecation after surgery	5.16		*p =* 0.023
	Quality of life assessment [Mean ± SD]	anxiety level	at 24h after surgery	11.17 ± 1.34	10.61 ± 0.93	*p =* 0.078
			at 48h after surgery	10.94 ± 1.22	10.64 ± 0.93	*p =* 0.344
		eating and sleeping	at 24h after surgery	8.67 ± 1.10	9.06 ± 1.84	*p =* 0.532
			at 48h after surgery	8.67 ± 1.17	8.92 ± 1.92	*p =* 0.980

a *The studies reported there was no significant difference on sex, age, hemorrhoid stage and pain before surgery, etc. at baseline between the two groups (all p > 0.05), but did not provide pain level data at baseline*;

b *The study reported that the change of VRS and WBS was analyzed by Cochran-Mantel-Haenszel method*.

*VAS, visual analog scale; VRS, verbal rating scale; WBS, Wong-Baker Faces scale*.

#### Acupuncture vs. Sham Acupuncture

Wu's study (30) (*n* = 72) compared EA with SA. The treatment was initiated 15 min after the surgery and lasted for 30 min. Patients' pain levels were measured using VAS, VRS, and WBS score on an hourly basis in the first 8 h after surgery, and then once daily until 48 h (10 times in total), and at the first defecation after surgery, respectively.

The study reported that the VAS score was declining gradually in both groups after surgery, but was significantly lower in the EA group compared to that of the SA group at 6 h (2.11 ± 0.85 vs. 2.49 ± 0.58, *p* = 0.033), 24 h (1.08 ± 0.66 vs. 1.51 ± 0.66, *p* = 0.006) after surgery and during the first defecation (1.22 ± 0.64 vs. 1.89 ± 0.88, *p* < 0.001). Similarly, the EA group saw a significant lower level of VRS score at 4 h after surgery (χ^2^ = 4.47, *p* = 0.035) and during first defecation (χ^2^ = 6.00, *p* = 0.014), and WBS score at 5, 7, and 8 h after surgery (χ^2^ = 5.69, 6.44, 6.04, *p* = 0.017, 0.011, 0.014, respectively), and during first defecation (χ^2^ = 5.16, *p* = 0.023), compared to the SA group. However, the between-group differences of VAS, VRS, and WBS were not significant at other time points measured. The study also measured the quality of life using the anxiety subscale, diet, and sleep subscale from the Symptom Checklist-90 Scale between the two groups at 24 and 48 h after surgery; however, no significant difference was found regarding anxiety, eating, and sleeping between the two groups (*p* > 0.05).

In addition, the study neither report any use of rescue analgesic drugs adjunct to acupuncture treatment nor any information on the time for incision healing, length of hospital stay, and effect at longer time point, etc.

#### Acupuncture vs. Active Medication

Three studies (20, 28, 29) compared the effect of electroacupuncture or MA with active medicines for PHP, such as tramadol (a non-opioid central analgesic drug) (29), methylene blue plus bupivacaine hydrochloride injection (a topical anesthetic) (28), and indomethacin suppositories (a non-steroidal anti-inflammatory drugs [NSAIDs]) (20). Xiao's study (20) (*n* = 80) reported that the MA group had a significant lower level of VAS score than that of indometacin suppositories group at the 1st (5.75 ± 1.04 vs. 6.18 ± 1.12, *p* = 0.0395), 3^rd^ (3.87 ± 1.09 vs. 5.46 ± 1.14, *p* < 0.001), 5th (3.05 ± 1.13 vs. 4.63 ± 1.07, *p* < 0.001) and 7th (2.01 ± 0.96 vs. 4.08 ± 0.79, *p* < 0.001) days after surgery, but no data of pain level within 24 h and during defecation was reported. Lin's study (29) (*n* = 58) reported VAS score within 72 h after surgery (at 2, 4, 10, 24, 48, and 72 h, respectively), and found the level of VAS score at 48 h after surgery in MA group was significantly lower than that in tramadol hydrochloride group (2.10 ± 0.90 vs. 2.41 ± 1.12, *p* = 0.046); however, no significant difference on VAS score was found between the two groups at other time points. Similarly, this study did not mention any data of pain level during defecation.

Zeng's study (28) (*n* = 65), which also used EA for PHP, focused on pain level during the defecation, and found that the VAS scores in EA group were significantly lower than that in the control group (using bupivacaine hydrochloride as topical anesthetics) at the 1st (5.33 ± 1.43 vs. 7.03 ± 1.66, *p* < 0.001), 2nd (4.67 ± 1.79 vs. 7.72 ± 0.88, *p* < 0.001), 3^rd^ (3.61 ± 1.78 vs. 6.14 ± 1.41, *p* < 0.001), 5th (2.44 ± 1.86 vs. 4.69 ± 1.63, *p* < 0.001) and 7th (1.61 ± 1.50 vs. 3.17 ± 1.73, *p* = 0.003) days after surgery.

Lin's study (29) also reported the patient's use and dose of rescue analgesic drugs (tramadol hydrochloride tablets) when the analgesic effect of acupuncture was unsatisfactory. It showed that the per capita dose of analgesic drug in MA group was significantly lower than that in the control group (50.00 ± 0.00 vs. 167.24 ± 30.69, *p* = 0.003, unit mg). However, the other two studies under this category did not report any information on the use of rescue analgesic drugs. None of the three studies provided any outcome data at a longer follow-up time.

#### Adverse Events

Among the four studies, one study (29) reported dizziness (1/29), nausea (1/29), urination disorder (2/29), and constipation (1/29) in the acupuncture group. One study (28) reported the urine retention (2/35) and constipation (1/35). Two studies (20, 30) reported no adverse events in the acupuncture group. No serious adverse events were reported in any of the included studies.

## Discussion

The four studies included in this systematic review showed that both EA and MA can relieve PHP at various time points and defecation after hemorrhoidectomy, compared with SA or active medication. By comparing with SA, Wu's study (30) demonstrated a net effect of EA on reducing pain level after hemorrhoidectomy, especially within the first day and defecation after surgery. Zeng's study (28) also demonstrated a significant lower level of pain during defecation in the EA group compared with active medication up to 1 week after surgery. However, the two studies using MA reported inconsistent results on the effect of pain relief after hemorrhoidectomy. Substantial variation was found on the risk of bias level across included studies.

A review (32) of mechanisms of acupuncture on persistent pain revealed that EA can alleviate both sensory and affective inflammatory pain by activating a variety of bioactive chemicals through peripheral, spinal, and supraspinal mechanisms. It also suggested that EA, when combined with low dosages of conventional analgesics, provides effective pain management that can reduce the side effects of analgesic drugs. The above-mentioned evidence brought explanatory support to the beneficiary effect observed in the two studies adopting EA for PHP. And it is worth noticing that the two study on EA demonstrated consistently that EA can significantly reduce pain level during defecation up to 7 days after surgery, either compared with SA or topical anesthetics, and this might be an advantage of EA where conventional analgesics was not applicable due to side effect or other reasons. The differences in VAS score during defecation at various time points after surgery between EA and control groups in Zeng's study were more than 1.5 points on average, and exceeded the minimum clinically important difference (MCID) of one point on VAS summarized by a study (33) measuring acute postoperative pain. In Zeng's study, the difference in VAS score at first defecation after surgery was comparable to data reported by a systematic review (34) on metronidazole for PHP, and the difference in VAS score during defecation at 1 week after surgery was also comparable to that reported by a recent study on mesoglycan for PHP compared with standard post-operative therapy (ketorolac tromethamine) (35). However, the difference in VAS score during the first defecation after surgery in Wu' study was <1 point and not clinically important.

In addition, higher current frequencies were adopted in Wu's (50 Hz) and Zeng's study (50 Hz) compared with data from previous review (32), which suggested that EA inhibits inflammatory and neuropathic pain more effectively at 2–10 Hz than that at 100 Hz. And the two studies were using different acupoints, and intensities of electric current were not specified. Such discrepancy may need further investigation to figure out an appropriate and standardized range of electric current frequency and intensity, as well as the selection of optimal acupoints.

Although some studies (32, 36, 37) proved that EA produced higher pain threshold elevation than MA, indicating that MA may not as effective as EA on pain relief; another study (21) showed that MA was an effective complementary to NSAIDs compared with NSAIDs plus SA in the treatment of post-tonsillectomy pain measured by VAS score. However, the observation was ceased at 3 h after the surgery. One study (20) in this review, using indomethacin suppositories (a type of NSAIDs) as comparison, found a longer effect (up to 7 days) of MA on PHP; however, it was lack of SA as comparison to estimate its net effect size. When using tramadol hydrochloride as comparison, it seemed that the superior effect of MA decreased, as the difference on VAS score between the two groups was found significant only at 48 h after hemorrhoidectomy (29). Another factor that may lead to over-estimation of the effect size of MA was the time that conventional analgesic drugs were administered. Both indomethacin suppositories (20) and tramadol hydrochloride (29) were administered after surgery in the two studies; whereas, it was recommended that these drugs should be taken as early as in preoperative or intraoperative period to provide sufficient analgesic effect in the early recovery period (7). Further, it was interesting that the VAS score measured in Xiao's study was generally higher than that in Lin's study at the same time points. As the VAS score can be subjected to measurement bias and the risk of bias was high in the two studies on MA, the VAS score reported by the two studies was questionable, likely contributing to the disparities found between the two studies. In addition, although Lin's study showed that acupuncture reduced the per capita dose of rescue analgesic drug use, the effect is suspicious due to the small sample size and high risk of bias.

Only Milligan–Morgan procedure (open hemorrhoidectomy) was discussed in all included studies. Other types of hemorrhoidal surgeries, such as Ferguson procedures (commonly used closed hemorrhoidectomy) and hemorrhoidopexy, were not reflected by any studies included in this review. These procedures were reported to be associated with less postoperative pain, faster wound healing, lesser risk of postoperative bleeding, and longer procedure time according to the recent meta-analysis (38, 39). Therefore, it is not possible to know whether the effect of acupuncture detected in this review can be generalized to other population experiencing different types of hemorrhoidal surgeries.

It was hard to know whether the on-set of pain relief effect of the acupuncture (both EA and MA) was prior to active medication with data available from included studies; however, it seemed that the effect of acupuncture can last overtime to relieve pain during defecation, of which the time was not predictable. Nevertheless, there was no follow-up data beyond 1 week to demonstrate the effect of both EA and MA on PHP in a longer period, as the recovery time varies from 1 to 4 weeks or even beyond according to different types of surgeries and treatments (8, 40, 41), as well as data of other outcomes on post-operative bleeding, activity, change to quality of life, length of hospital stay, etc. In terms of adverse events reported, data were not adequate to determine whether the adverse event was associated with acupuncture or not.

## Limitations

Limited by human resources and capacity, we were not able to search and include papers published in languages other than English and Chinese. Due to a small number of studies included, and lack of information on anesthesia technique, duration and extent of surgery, preexisting pain levels, perioperative opioid levels, and severity degree of hemorrhoids (grade I–IV), we were not able to conduct subgroup analyses by the types of hemorrhoidectomies, types of analgesics, severity degree of hemorrhoids, acupoints, etc. The level of risk of bias across included studies varied largely and could undermine the validity of the effect size observed in this review. In addition, all included studies were conducted in China; therefore, the generalizability of the result to other population was constrained.

## Conclusions

Although the benefit of acupuncture on PHP, especially EA on defecation after surgery, was observed at some time points, evidence on the effectiveness of both EA and MA was not conclusive due to a very limited number of studies and substantial factors that could undermine the effect size of acupuncture on PHP and prevent the generalizability of the result. To further investigate the true effect size and safety of acupuncture compared with different conventional medications and their contributing factors, well-designed RCTs with large sample sizes, standardized treatment regimen, and outcome measures are necessary.

## Data Availability Statement

The original contributions presented in the study are included in the article/Supplementary Material, further inquiries can be directed to the corresponding author.

## Author Contributions

HC and ZL contributed to the project development. RJ, WZ and YS contributed to the database searches, literature screening, and data extraction. WZ and HC contributed to the data analysis and the manuscript writing. ZL contributed to the critical review of the manuscript. All the authors approved the final version of this manuscript to be published and agree to be accountable for all the aspects of this study.

## Conflict of Interest

The authors declare that the research was conducted in the absence of any commercial or financial relationships that could be construed as a potential conflict of interest.

## Publisher's Note

All claims expressed in this article are solely those of the authors and do not necessarily represent those of their affiliated organizations, or those of the publisher, the editors and the reviewers. Any product that may be evaluated in this article, or claim that may be made by its manufacturer, is not guaranteed or endorsed by the publisher.

## Abbreviations

PHP: post-hemorrhoidectomy pain
RCTs: randomized controlled trials
VAS: visual analog scale
VRS: verbal rating scale
WBS: Wong-Baker Faces scale
EA: electro-acupuncture
SA: sham acupuncture
NSAIDs: nonsteroidal anti-inflammatory drugs
PRISMA: Preferred Reporting Items for Systematic Review and Meta-Analysis
MD: mean difference
CI: confidence interval
TCM: traditional Chinese medicine
CNKI: China National Knowledge Infrastructure
CBM: Chinese Biomedical database
VIP: Chinese Scientific Journal Database.
