# Supplementary material for: The Role of Acupuncture in Relieving Post-Hemorrhoidectomy Pain: A Systematic Review of Randomized Controlled Trials
Source: Front Surg. 2022 Mar 28;9:815618. doi: 10.3389/fsurg.2022.815618 (PMC8995644; doi:10.3389/fsurg.2022.815618)
Supplement: Supplementary file 1 [file Data_Sheet_1.PDF]

Appendix A. Search strategy

The detailed search strategies are provided below.

	**Pub Med: search items**	Results
1	Pain, Postoperative. MeSH	39074
2	Postoperative pain. ti,ab	24226
3	Postoperative pains. ti,ab	38
4	Pain after surgery. ti,ab	701
5	Pain after operation. ti,ab	4084
6	post-operative pain. ti,ab	3035
7	or 1-6	54264
8	pain. ti,ab	593936
9	ache. ti,ab	15619
10	soreness. ti,ab	3155
11	or 8-10	611224
12	Postoperative Period. MeSH	53052
13	Postoperative. ti,ab	450951
14	or 12-13	477451
15	11 and 14	64164
16	or 7,15	86728
17	Hemorrhoids. MeSH	5102
18	hemorrhoids. ti,ab	3235
19	hemorrhoid. ti,ab	630
20	piles. ti,ab	1386
21	or17-20	7288
22	16 and 21	874
23	Acupuncture. MeSH	24162
24	acupuncture. ti,ab	21117
25	electroacupuncture. ti,ab	4195
26	galvano-acupuncture. ti,ab	2
27	electric acupuncture. ti,ab	75
28	needling. ti,ab	2757
29	needle. ti,ab	100710
30	or 23-29	128957
31	randomized controlled trial. Pt	492614
32	Randomized Controlled Trials as Topic. MeSH	130251
33	Controlled Clinical Trial. ti,ab	13476
34	controlled trial. ti,ab	122343

35	randomized. ti,ab	494952
36	randomly. ti,ab	320548
37	placebo. ti,ab	207201
38	groups. ti,ab	1992600
39	or 31-38	2706714
40	22 and 30 and 39	22

	**Cochrane: search items**	Results
1	Pain, Postoperative. MeSH	14129
2	Postoperative pain. ti,ab,kw	40565
3	Postoperative pains. ti,ab,kw	40558
4	Pain after surgery. ti,ab,kw	30627
5	Pain after operation. ti,ab,kw	14976
6	post-operative pain. ti,ab,kw	7686
7	or 1-6	51765
8	pain. ti,ab,kw	164904
9	ache. ti,ab,kw	1336
10	soreness. ti,ab,kw	5208
11	or 8-10	169110
12	Postoperative Period. MeSH	5657
13	Postoperative. ti,ab,kw	113531
14	or 12-13	114484
15	11 and 14	41464
16	or 7,15	52626
17	Hemorrhoids. MeSH	592
18	hemorrhoids. ti,ab,kw	1427
19	hemorrhoid. ti,ab,kw	1427
20	piles. ti,ab,kw	171
21	or17-20	1498
22	16 and 21	703
23	Acupuncture. MeSH	141
24	acupuncture. ti,ab,kw	13718
25	electroacupuncture. ti,ab,kw	2295
26	galvano-acupuncture. ti,ab,kw	3
27	electric acupuncture. ti,ab,kw	1147
28	needling. ti,ab,kw	14748
29	needle. ti,ab,kw	14750
30	or 23-29	26301

31	randomized controlled trial. Pt	478882
32	Randomized Controlled Trials as Topic. MeSH	13966
33	Controlled Clinical Trial. ti,ab,kw	506360
34	controlled trial. ti,ab,kw	654350
35	randomized. ti,ab,kw	929618
36	randomly. ti,ab,kw	869305
37	placebo. ti,ab,kw	286671
38	groups. ti,ab,kw	697096
39	or 31-38	1239662
40	22 and 30 and 39	31

	**Embase: search items**	Results
**1**	'Pain, Postoperative'. exp OR 'Pain, Postoperative'	63490
**2**	'Postoperative pain'. exp	57659
**3**	'Postoperative pains'.tw	61
**4**	'Pain after surgery'.tw	941
**5**	'Pain after operation'.tw	109
**6**	'post-operative pain'.tw	5363
**7**	pain.tw	1117017
**8**	ache.tw	18421
**9**	soreness.tw	3514
**10**	Soreness. exp	23
**11**	Pain. exp	1138274
**12**	Postoperative.tw	910710
**13**	'Postoperative Period'.tw	201042
**14**	'Postoperative analgesia'. exp	14247
**15**	'Postoperative Period'. exp	437420
**16**	#12 OR #13 OR #15	1058864
**17**	#7 OR #8 OR #9 OR #10 OR #11	1428857
**18**	#16 AND #17	175355
**19**	#1 OR #2 OR #3 OR #4 OR #5 OR #6 OR #14 OR #18	179867
**20**	haemorrhoid.tw	4258
**21**	haemorrhoids.tw	9352
**22**	hemorrhoid. exp	9032
**23**	haemorrhoid.tw	283
**24**	haemorrhoids.tw	1887
**25**	piles.tw	1778
**26**	#20 OR #21 OR #22 OR #23 OR #24 OR #25	12026
**27**	acupuncture.tw	44440

**28**	acupuncture. exp	41520
**29**	electroacupuncture.tw	6405
**30**	'galvano acupuncture'.tw	1
**31**	'electric acupuncture'.tw	149
**32**	needling.tw	179361
**33**	needle.tw	3219
**34**	'acupuncture needle'. exp	834
**35**	#27 OR #28 OR #29 OR #30 OR #31 OR #32 OR #33 OR #34	222543
**36**	'randomized controlled trial'.tw	638819
**37**	'randomized controlled trial'. exp	490728
**38**	'randomized controlled trial (topic)'. exp	138697
**39**	'controlled clinical trial'.tw	441028
**40**	'controlled trial'.tw	661355
**41**	randomized.tw	917594
**42**	randomly.tw	317656
**43**	placebo.tw	412520
**44**	groups.tw	2470946
**45**	#36 OR #37 OR #38 OR #39 OR #40 OR #41 OR #42 OR #43 OR #44	3531699
**46**	#19 AND #26 AND #35 AND #45	13

	**CNKI: search items**	Results
**#1**	((SU='针'+'电针'+'针刺'+'针灸') or (TI='针'+'电针'+'针刺'+'针灸') or (AB='针'+'电针'+'针刺'+'针灸') and FT='随机') and ((SU=('痔'+'痔疮'+'肛肠病'+'肛周'+'直肠') *'术后') or (TI=('痔'+'痔疮'+'肛肠病'+'肛周'+'直肠') *'术后') or (AB=('痔'+'痔疮'+'肛肠病'+'肛周'+'直肠') *'术后') and (FT='疼痛'+'镇痛'+'止疼'+'止痛'+'麻醉'))	403

	**VIP: search items**	Results
**#1**	任意字段=随机对照 或者 任意字段=对照 或者 任意字段=对比 或者 任意字段=比较 并且 任意字段=针 或者 任意字段=针刺 或者 任意字段=电针 或者 任意字段=针灸 并且 任意字段=疼痛 或者 任意字段=镇痛 或者 任意字段=麻醉 或者 任意字段=止疼 或者 任意字段=止痛 并且 任意字段=痔疮 或者 任意字段=肛肠病 或者 任意字段=肛周 或者 任意字段=直肠 并且 任意字段=术后	159

	**CBM: search items**	Results
**#1**	("痔疮"[摘要:智能] OR "肛肠病"[摘要:智能] OR "肛周"[摘要:智能] OR "直肠"[摘要:智能]) AND "术后"[摘要:智能] AND ("疼痛"[全字段:智能] OR "镇痛"[全字段:智能] OR "止疼"[全字段:智能] OR "止	231

	痛"[全字段:智能] OR "麻醉"[全字段:智能]) AND ("针"[摘要:智能] OR "针灸"[摘要:智能] OR "针刺"[摘要:智能] OR "电针"[摘要:智能]) AND ("随机对照"[全字段:智能] OR "对照"[全字段:智能] OR "对比"[全字段:智能] OR "比较"[全字段:智能])	

	**Wanfang: search items**	Results
**#1**	（摘要:(痔疮)+摘要:(肛肠病)+摘要:(肛周)+摘要:(直肠)）*摘要:(术后)*(摘要:(疼痛)+摘要:(镇痛)+摘要:(麻醉)+摘要:(止疼)+摘要:(止痛))*(摘要:(针)+摘要:(针刺)+摘要:(针灸)+摘要:(电针))*(摘要:(随机对照)+摘要:(对照)+摘要:(对比)+摘要:(比较)+摘要:(随机))	136

	**Medical Online: search items**	Results
**#1**	「鍼療法(タイトル) OR 鍼療法(キーワード) OR 鍼療法(アブストラクト) OR 鍼(タイトル) OR 鍼(キーワード) OR 鍼(アブストラクト) OR 鍼灸医学(タイトル) OR 鍼灸医学(キーワード) OR 鍼灸医学(アブストラクト) OR acupuncture(タイトル) OR acupuncture(キーワード) OR acupuncture(アブストラクト)」	10706
**#2**	「術後の痛み(タイトル) OR 術後の痛み(キーワード) OR 術後の痛み(アブストラクト) OR 手術後痛み(タイトル) OR 手術後痛み(キーワード) OR 手術後痛み(アブストラクト)」	383
**#3**	「hemorrhoidectomy(タイトル) OR hemorrhoidectomy(キーワード) OR hemorrhoidectomy(アブストラクト) OR 痔疾(タイトル) OR 痔疾(キーワード) OR 痔疾(アブストラクト) OR 手術し痔(タイトル) OR 手術し痔(キーワード) OR 手術し痔(アブストラクト) OR 痔(タイトル) OR 痔(キーワード) OR 痔(アブストラクト)」	1332
**#4**	「ランダム化比較試験(タイトル) OR ランダム化比較試験(キーワード) OR ランダム化比較試験(アブストラクト) OR 無作為対照試験(タイトル) OR 無作為対照試験(キーワード) OR 無作為対照試験(アブストラクト)」	3555
**#5**	#1 and #2 and #3 and #4	0

	**Korea Science: search items**	Results
**#1**	침 OR "침술" OR "침구" OR acupuncture	4606
**#2**	("hemorrhoid" OR "치질") AND "수술" AND ("아프" OR "pain")	4
**#3**	"무작위 비교 실험" OR "randomized controlled trial"	18
**#4**	#1 and #2 and #3	0
